# Acneiform Eruption Secondary to Over-the-Counter Vitamin B12

**DOI:** 10.7759/cureus.43275

**Published:** 2023-08-10

**Authors:** Acacia Bowden, Odera Ekeh, Nicholas D Brownstone, Sylvia Hsu

**Affiliations:** 1 Dermatology, University of Rochester School of Medicine and Dentistry, Rochester, USA; 2 Dermatology, Cooper Medical School of Rowan University, Camden, USA; 3 Dermatology, Temple University Hospital, Philadelphia, USA

**Keywords:** medical dermatology, b vitamins, clinical dermatology, vitamin b12, acneiform eruption

## Abstract

Treating an acneiform eruption requires the discovery of its etiology. Often, the removal of the offending agent can lead to the resolution of the eruption, resulting in an excellent prognosis. Herein, we present a rare case of a vitamin B12-induced acneiform eruption occurring in a 68-year-old female due to an over-the-counter supplement.

## Introduction

Acneiform eruptions are a group of skin disorders that resemble acne vulgaris with characteristic abrupt-onset and pruritic monomorphous erythematous papules, pustules, and/or nodules that are generally found on the chest and back. Drugs known to induce acneiform eruptions include corticosteroids, antituberculosis drugs, anticonvulsants, antipsychotics, and some anticancer agents [1,2]. Some dietary supplements have also been linked to acneiform eruptions. There are only a handful of reported cases in the literature of acneiform eruptions due to vitamin B12 [3-5].

## Case presentation

**Figure 1:**
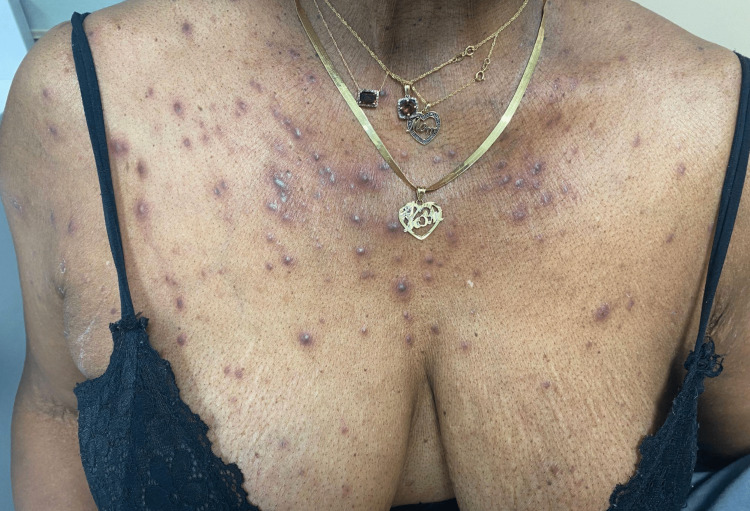
Monomorphic eruption of erythematous papules, pustules, and nodules on the chest.

A 68-year-old female presented with abrupt-onset and pruritic eruption lasting two weeks. Physical examination revealed monomorphic erythematous papules and pustules on the face, chest (Figure 1), arms, and back. The patient has a past medical history of hypertension, cervical stenosis, and tobacco use disorder. The patient denied any new medications. Her current medications include amlodipine, and she had been on the same stable dose for many years. Upon asking about over-the-counter medications, we found that the patient recently started taking over-the-counter vitamin B12 weekly for the past 1-2 months. Amlodipine is not known to cause acneiform drug eruption; thus, the vitamin B12 was determined to be the cause. She was treated with a course of doxycycline. We recommended that she continue the vitamin B12 repletion as per the recommendation of her primary care provider.

## Discussion

Acneiform eruptions are known to occur secondary to many drugs, commonly including corticosteroids, phenytoin, olanzapine, lithium, isoniazid, thiourea, thiouracil, disulfiram, corticotropin, nystatin, itraconazole, hydroxychloroquine, naproxen, mercury, amineptine, chemotherapy drugs, and epidermal growth factor receptor inhibitors. Antibiotics, such as penicillins, macrolides, co-trimoxazole, fluoroquinolones, and chloramphenicol, have also been known to provoke acneiform eruptions [1]. However, acneiform eruptions due to vitamin B12 have rarely been described in the literature, and of those reported, most of the patients have been female [2]. Patients are typically counseled on transient chromaturia, increased blood pressure, nausea, and headache as adverse side effects of vitamin B12, but with so few cases, vitamin B12-induced acneiform eruptions often go unmentioned [2].

There are only 39 cases of vitamin B12-induced acneiform eruption. Ranging from 17 to 62 years old, patients presented with acneiform eruptions between one day and five months following vitamin B12 initiation. The majority of the patients (26/39) were females, and most (22/39) received intramuscular hydroxycobalamin [3-5]. In our patient, she was taking vitamin B12 orally as an over-the-counter supplement for 1-2 months per her primary care physician’s recommendation.

The pathophysiology behind vitamin B12-induced acneiform eruption is not understood, but recent studies demonstrate increased levels of vitamin B12 in the pilosebaceous follicle. Vitamin B12 supplementation leads *Cutibacterium acnes* to stimulate the production of porphyrins that, through oxidation on the skin surface, results in the release of pro-inflammatory substances that prime the environment for acneiform lesions [6]. Unlike acne vulgaris, however, acneiform eruption lesions are often non-scarring and resolve within two to three weeks after the cessation of the causative agent.

## Conclusions

Acneiform eruptions are rarely reported to be caused by vitamin B12. In this case, when the patient’s prescribed medication history did not reveal the probable cause of the acneiform eruption, further questioning revealed her intake of over-the-counter vitamin B12 supplements. Thus, when determining the etiology of acneiform eruptions, clinicians should ask about any medications or supplements a patient might be taking whether prescribed or over-the-counter.
